# Supplementary material for: Pharmacologic ROMK Inhibition Protects Against Myocardial Ischemia Reperfusion Injury
Source: Int J Mol Sci. 2025 Apr 17;26(8):3795. doi: 10.3390/ijms26083795 (PMC12028082; doi:10.3390/ijms26083795)
Supplement: Supplementary file 1 [file ijms-26-03795-s001.zip › ijms-3500443-supplementary.pdf]

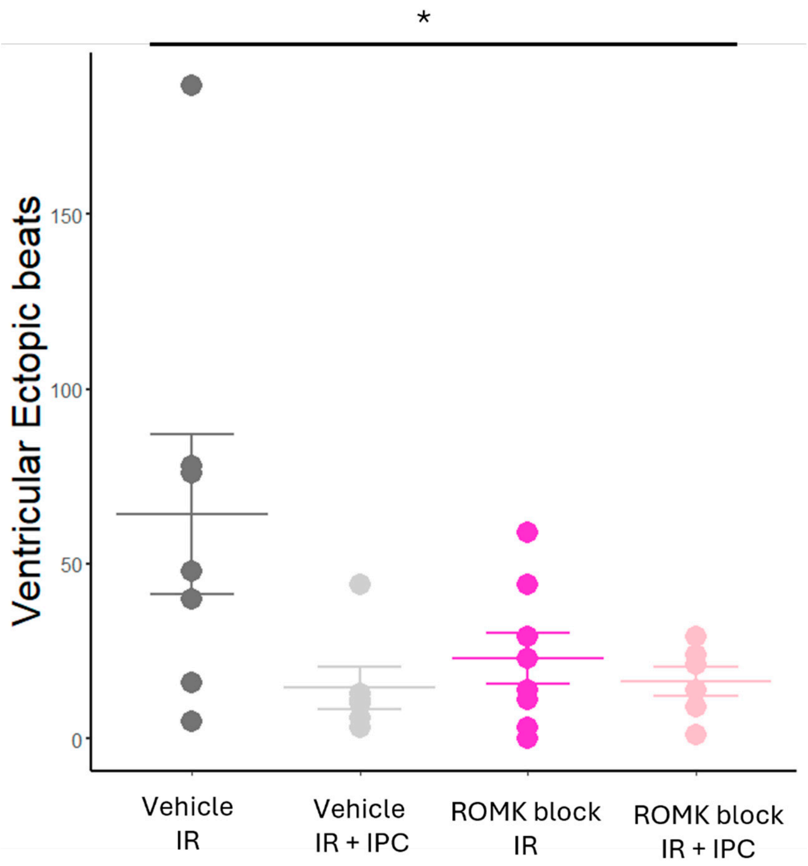

Figure S1. Ventricular ectopic beats in the first 5 minutes of reperfusion. $^{*}P<0.05$. One-way ANOVA was performed.

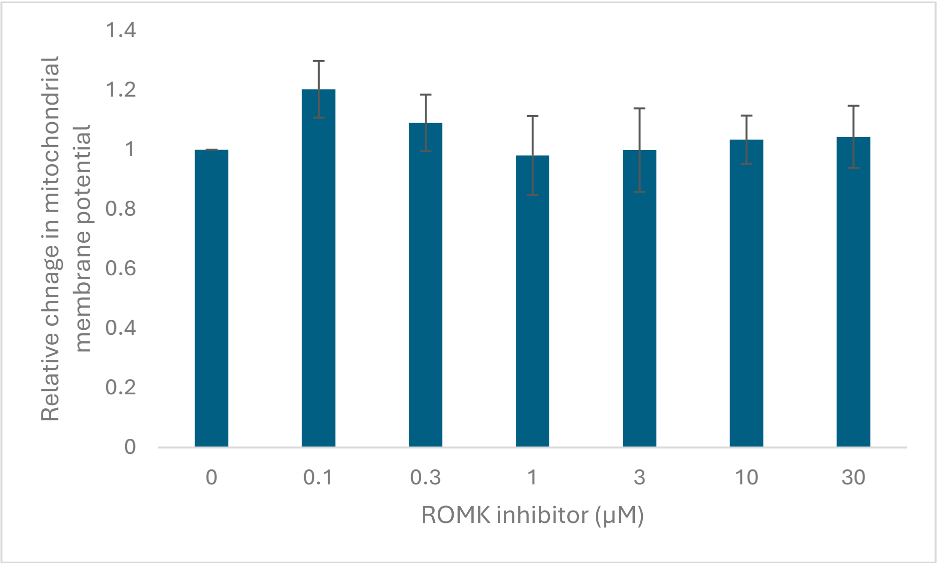

Figure S2. Change in mitochondrial membrane potential from ATP after ROMK inhibition. The change in adult murine isolated cardiac mitochondrial membrane potential from the addition of

1000 µM of ATP was measured by rhodamine florescence. The addition of the ROMK inhibitor did not significantly block the effect of ATP hyperpolarization.

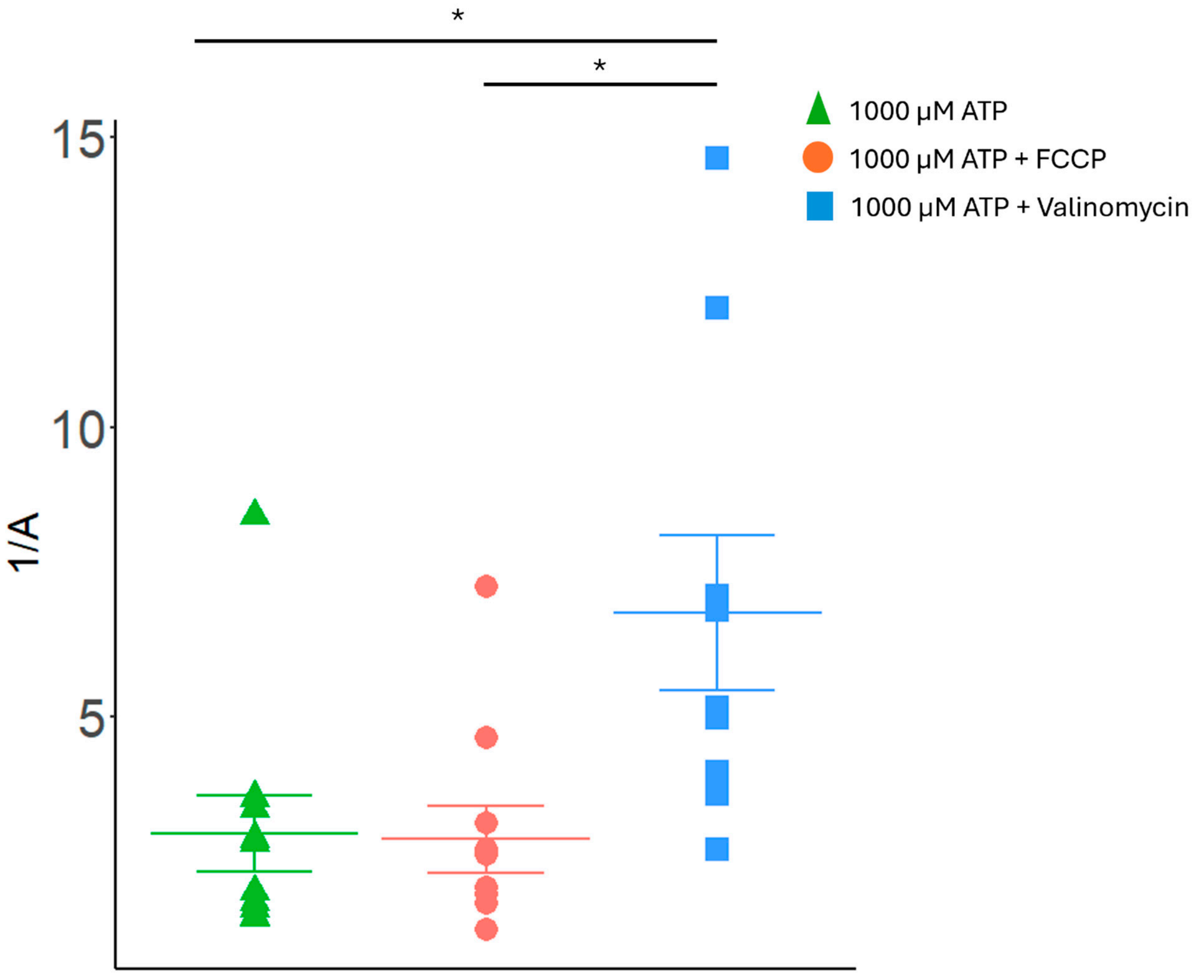

Figure S3. The effect of FCCP and Valinomycin on the swelling of isolated mitochondria. The swelling of isolated adult murine cardiac mitochondria was assessed by light scattering at 540 nm using a spectrophotometer. Mitochondria were measured in the presence of 1000 µM of ATP and either the addition of the K+ ionophore Valinomycin 2 nM or the H+ ionophore FCCP 1 µM. As expected the treatment of K+ ionophore Valinomycin greatly increased the scattering of light and swelling of mitochondria from the influx on K+ and the subsequent influx of H_2O. *,P<0.01. One-way ANOVA was performed with the post hoc Tukey HSD test to assess differences between groups.
